# Dead-Space Microdomains as Explicit Barriers to Extracellular Drug Transport in Alzheimer’s Disease

**DOI:** 10.1007/s11095-026-04129-w

**Published:** 2026-06-09

**Authors:** Dimitrios Charemis, Dimitrios Lampropoulos, Yannis Dimakopoulos, Gregory Sivolapenko, Maria Hadjinicolaou

**Affiliations:** 1 School of Science and Technology, Hellenic Open University, P. Aristotelous 18, Patra, Achaia Greece; 2 Department of Chemical Engineering, University of Patras, Caratheodory 1, University Campus, Patra, Achaia Greece; 3 Department of Pharmacy, University of Patras, Caratheodory 1, University Campus, Patra, Achaia Greece

**Keywords:** CNS drug delivery, Alzheimer’s disease, Dead-space microdomains, Neuropharmaceuticals

## Abstract

**Objective:**

The impact of dead-space (DS) microdomains on cerebral drug penetration has been demonstrated experimentally; yet computational models typically capture their effects indirectly through effective tortuosity parameters. Here, we develop an anatomically grounded framework that systematically evaluates pharmaceutical diffusion within the central nervous system (CNS).

**Methods:**

A finite element (FE) model was developed with DS explicitly represented as impermeable obstructions in the extracellular space (ECS) – consistent with structural remodeling observed in Alzheimer’s disease (AD). The model was calibrated by reproducing experimental diffusion timescales and subsequently applied to quantify the geometric contribution of DS to pharmaceutical transport.

**Results:**

Transport hindrance exhibited a strong dependence on molecular size. Within a representative ECS unit, the arrival of the $0.5c_0$ concentration contour at the venule was delayed by 11 s for Memantine, 14 s for Donepezil, and 80 s for Aducanumab. Spatial analyses revealed penetration delays that were not captured by domain-averaged uptake metrics. A Péclet number analysis confirmed that ECS transport for all three compounds remains diffusion-dominated under neurodegenerative conditions.

**Conclusions:**

The proposed framework provides a computationally efficient foundation for predictive multiphysics modeling in the human CNS and demonstrates how local ECS obstructions can lead to therapeutic hindrance in AD.

## Introduction

Quantitative modeling of solute transport in the brain’s extracellular space (ECS) is essential for studying the mechanisms governing neuropharmaceutical delivery and distribution. The ECS comprises approximately 20% of brain volume and forms an interstitial reticulum that mediates cellular communication and the distribution of neuroactive solutes [1–3]. Once viewed as relatively homogeneous, the brain ECS is now recognized as a highly heterogeneous, dynamic microenvironment shaped by local cellular architecture and glial processes. In this environment, microstructural features can dominate transport behavior independently of global porosity or average diffusivity [4–6]. Aging and neurodegenerative disease exacerbate heterogeneities – which are modulated by the properties of the extracellular matrix (ECM) [7–10]. Transport hindrance properties become critical in the context of Alzheimer’s disease (AD), where alterations in ECS structure can impair drug access to target regions [11].

**Fig. 1:**
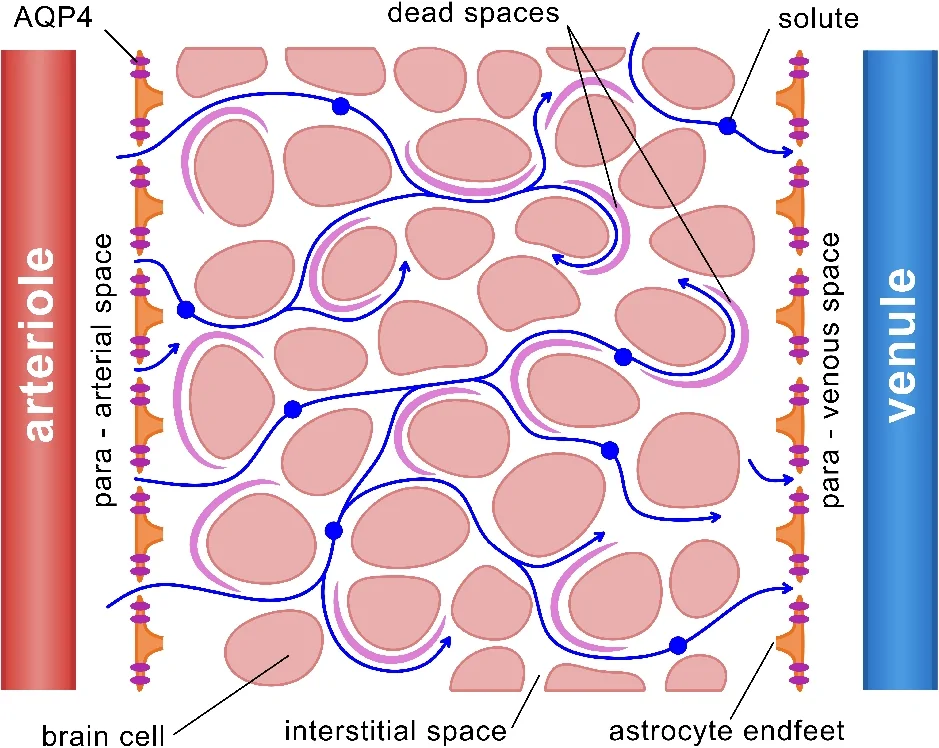
Schematic of the domain of interest used as a symmetric scaffold for ECS transport. Blue curves illustrate pathlines after the solute has entered the parenchyma through local capillaries, with gradient distortions shown near the DS. Adapted from [22]

A prominent source of microstructural heterogeneity is the presence of dead-space (DS) microdomains: semi-enclosed, disconnected cul-de-sac microenvironments that arise from abnormal cell packing configurations [12, 13]. Local causes of these microenvironments may be physiological but their role, frequency and spatial extent is amplified by pathological remodeling [14–16]. In AD, amyloid plaques deform ECS architecture, displace interstitial fluid and form barriers to diffusive transport [17]. Similar disruptions are observed adjacent to tumor ECM environments and within altered perivascular states after ischemic injury [18–20]. The presence of dead spaces (Fig. 1) indicates that successful BBB traversal does not preclude further transport hindrance, as secondary geometries may sequester solutes before they reach parenchymal targets [21].

Despite their physiological importance, dead spaces are rarely incorporated explicitly into computational models; instead, their influence is subsumed into effective tortuosity or porosity parameters that preserve global diffusion timescales and suppress local resolution [1, 23]. In this study, we decouple DS effects by transitioning to an explicit barrier analysis in which DS are represented as discrete geometric obstructions. While such microstructures are expected to impede solute penetration, their quantitative impact on concentration fields remains poorly characterized [24]. We argue that physiologically grounded representations of ECS heterogeneity are essential for predictive simulations [25], with direct implications for CNS drug development. Small-solute transport in parenchymal tissue is generally considered to be dominated by Fickian diffusion [26], although more general formulations may incorporate advection to account for large molecules [27–29]. We demonstrate that pathway-limited exposure metrics can diverge substantially from domain-averaged uptake, revealing spatially hidden underexposure in distal regions.

Our analysis proceeds in three stages. First, we calibrate the FE framework in a baseline geometry without dead spaces by reproducing experimentally reported timescales for a 3 kDa benchmark tracer (Tracer B, dextran) in healthy ECS tissue. Second, we introduce DS microdomains to the geometry and quantify how neurodegeneration redistributes diffusive flux and generates localized stagnation and penetration delays. Third, the DS framework is applied to three Alzheimer’s disease therapeutics to assess size-dependent vulnerability to microstructural obstruction.

**Table 1:** Geometrical parameters used in the FEM simulations

Symbol	Description	Value
*Porosity and thickness*		
$\varepsilon _{\textrm{p}}$	ECS porosity$^\text {a}$	0.20
λ	ECS (baseline) tortuosity$^\text {a}$	1.60
$\varepsilon _{\textrm{as}}$	Astrocyte layer porosity$^\text {b}$	$1.25\times 10^{-3}$
$th_{as}$	Astrocyte layer thickness$^\text {b}$	$1.0\times 10^{-6}$ m
$th_d$	Out-of-plane domain thickness	$2.5\times 10^{-4}$ m
$th_{pv}$	Para-venous space thickness	$3.1\times 10^{-8}$ m
$th_{pa}$	Para-arterial space thickness$^\text {c}$	$2.4\times 10^{-8}$ m
*Geometrical dimensions*		
$l_c$	Computational cell arm length	$2.5\times 10^{-4}$ m
$r_{ven}$	Venule radius$^\text {d}$	$2.0\times 10^{-5}$ m
$r_{art}$	Arteriole radius	$1.5\times 10^{-5}$ m
$r_{ds}$	Dead space radius	$5.0\times 10^{-6}$ m

$$^\text {a}$$ From [6].

$$^\text {b}$$ From [32].

$$^\text {c}$$ From [33].

$$^\text {d}$$ From [22]

## Materials & Methods

### Model Formulation

Prior studies have modeled the distribution of brain cells using ECM reconstructions. Here, global approximations for cell placement retain sufficient accuracy whilst substantially reducing computational times for porous media calculations [30]. By abstracting cell placement and focusing on DS and ECS architecture, we enable controlled comparisons between healthy and pathological conditions. This approach also improves mesh convergence, allowing for parameter sweeps and efficient approaches to iterative numerical simulation cycles [31].

Although capillaries are the predominant physiological route for solute entry into the ECS, our objective is not to model vascular exchange but to isolate the geometric hindrance effects of DS microdomains once solutes have already entered the brain parenchyma. Under linear diffusion, distributed microsources can be approximated by an equivalent boundary condition, allowing a symmetric scaffold with controlled input to reflect the key characteristics of extracellular transport. This approach builds upon [22] to provide a controlled assessment of DS-induced hindrance, independent of stochastic vascular distributions.

**Fig. 2:**
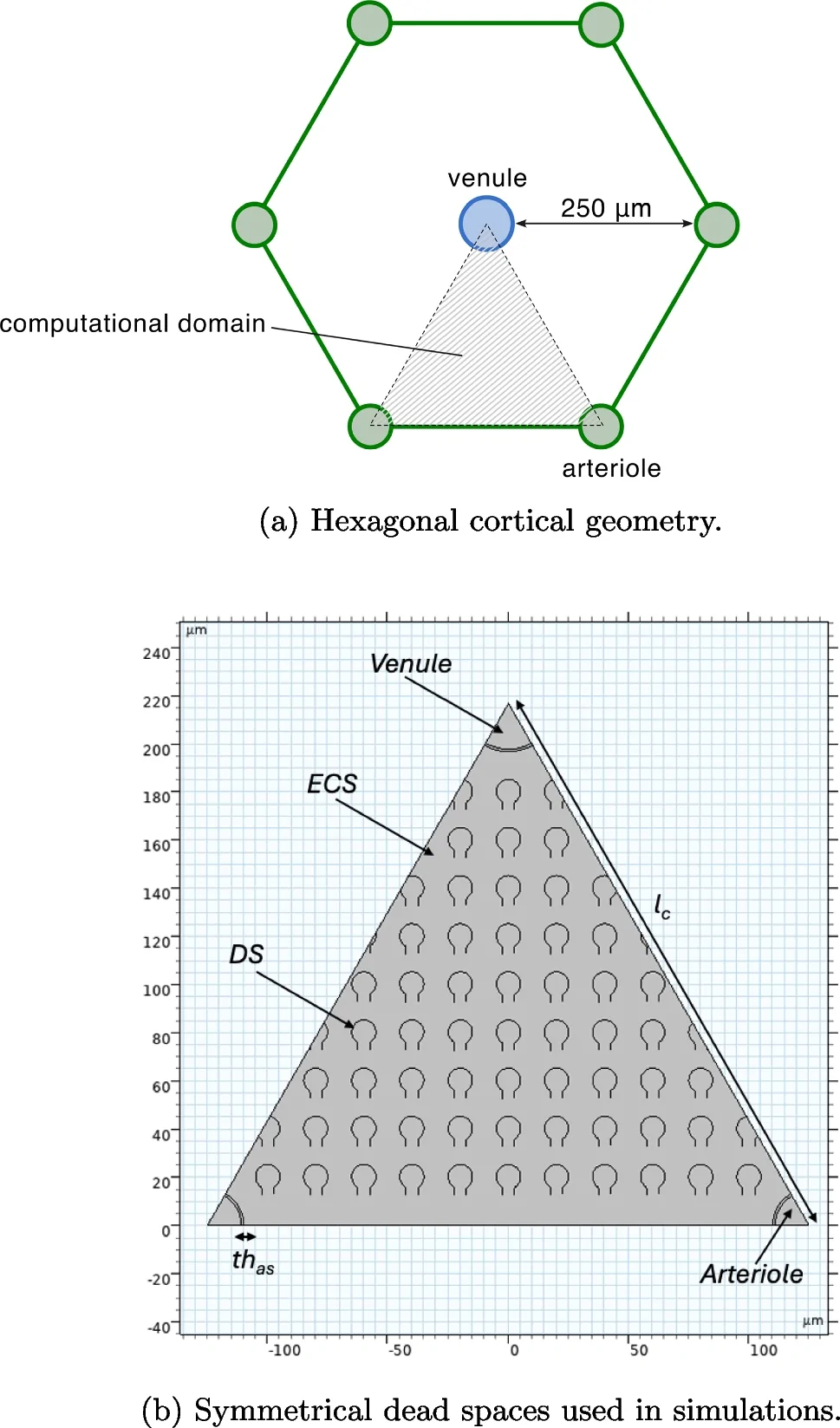
Computational domains used to simulate solute transport in the cortical ECS

Our finite element (FE) model tracks the concentration of a 3 kDa benchmark tracer (Tracer B), as well as three Alzheimer’s disease therapeutics – Memantine, Donepezil and Aducanumab – selected to span a range of molecular sizes and pharmacological classes. This selection allows our findings to generalize across the most common and emerging drug delivery strategies for AD. The geometrical parameters used in our model (Table 1) are anatomical and have been sourced from the literature.

The computational scaffold employed in this work consists of a 2:1 arteriole-to-venule ratio using a hexagonal unit; a central venule surrounded by six arterioles [34]. This geometry (Fig. 2a) replicates the microvascular topology in the human cortex, and can be tessellated to form three-dimensional domains. Domain symmetry (Fig. 2b) is exploited to optimize computational times while preserving transport behavior.

The dead-space radius of $r_{ds}=5.0\times 10^{-6}\ \textrm{m}$ is a mesoscale modeling abstraction representing ECS deformations and void regions arising from plaque deposition and altered cellular packing in early-stage AD [35]. The chosen value represents a conservative upper bound used to probe transport delays associated with large concave obstacles. Dead spaces are distributed stochastically in vivo [36]; we instead impose a symmetric spatial distribution to isolate mechanistic effects under conservative conditions. This configuration provides a lower-variability baseline against which spatial transport deficits can be identified without additional amplifications arising from clustering effects. See Appendix A for the mesh setup and mesh convergence analysis for the FEM simulation. See Appendix B for a sensitivity analysis on the radii and entrance sizes of DS.

### Governing Equations

We consider the enhanced convection–diffusion balance for a solute transported in the porous ECS with no sources or sinks. In this section, dimensional quantities are denoted by tildes. The governing equation is:

1 $$\begin{aligned} \varepsilon \,\frac{\partial \tilde{c}}{\partial \tilde{t}} +\tilde{\nabla }\cdot \left( \tilde{\textbf{u}}\,\tilde{c}\right) = \tilde{\nabla }\cdot \!\left( \tilde{\textbf{D}}^{*}\,\tilde{\nabla }\tilde{c}\right) , \end{aligned}$$

where $\tilde{c}$ is the solute concentration, ε is the ECS porosity and $\tilde{\textbf{u}}$ is the interstitial velocity. The effective diffusion tensor in the porous ECS is given by

2 $$\begin{aligned} \tilde{\textbf{D}}^{*} = \frac{\tilde{D}^{\textrm{free}}}{\lambda ^{2}}\,\textbf{I}, \end{aligned}$$

with $\tilde{D}^{\textrm{free}}$ the free aqueous diffusivity, λ the ECS tortuosity, and $\textbf{I}$ the identity tensor. See Appendix C for the characteristic scale analysis and the derivation of the dimensionless groups.

#### Final Dimensionless Governing Equation

Substituting the dimensionless groups and variables into Eq. 1 yields

3 $$\begin{aligned} \varepsilon \left( \frac{\tilde{c}_0}{\tilde{t}_c}\right) \frac{\partial c}{\partial t} +\left( \frac{\tilde{U}_c \tilde{c}_0}{\tilde{L}_c}\right) \nabla \cdot (\textbf{u}c) = \left( \frac{\tilde{c}_0 \tilde{D}^{\textrm{free}}}{\tilde{L}_c^2}\right) \nabla \cdot \!\left( \frac{1}{\lambda ^2}\nabla c\right) . \end{aligned}$$

Dividing through by the diffusive scaling factor $\tilde{c}_0 \tilde{D}^{\textrm{free}}/\tilde{L}_c^2$ gives the final dimensionless form:4

#### Neglecting Advection: Scaling Analysis

To investigate the importance of advective transport in the extracellular space (ECS), we perform a dimensional analysis based on the Péclet number, which quantifies the ratio of advective to diffusive transport rates:

5 $$\begin{aligned} \textrm{Pe} = \frac{\tilde{U}_c\,\tilde{L}_c}{\tilde{D}^{\mathrm {*}}}, \end{aligned}$$

where $\tilde{U}_c$ is a characteristic interstitial fluid velocity, $\tilde{L}_c$ is a characteristic length scale associated with microstructural obstructions (the spacing between dead-space microdomains), and $\tilde{D}^{\mathrm {*}}$ is the effective diffusivity of the solute in the ECS.

Experimental and modeling studies of parenchymal transport indicate that flow velocities in the neuropil are small due to the high hydraulic resistance of brain tissue. At distance from perivascular spaces, reported interstitial velocities are negligible or below $0.01~\mu \mathrm {m/s}$, with conservative upper-bound estimates of $\tilde{U}_c \approx 0.1~\mu \mathrm {m/s}$ [26, 37]. Adopting this upper bound together with a representative local microstructural length scale $\tilde{L}_c \approx 10~\mu \textrm{m}$ and an effective diffusivity typical of small-molecule therapeutics (e.g. Donepezil), $\tilde{D}^{\mathrm {*}} \approx 300~\mu \textrm{m}^2/\textrm{s}$, yields

6 $$\begin{aligned} \textrm{Pe} \approx \frac{(0.1~\mu \mathrm {m/s})(10~\mu \textrm{m})}{300~\mu \textrm{m}^2/\textrm{s}} \approx 3.3\times 10^{-3}\ll 1. \end{aligned}$$

Thus, transport in the parenchymal ECS lies firmly in the diffusion-dominated regime. Even if more conservative geometric scales are adopted (e.g. $\tilde{L}_c\sim 50~\mu \textrm{m}$ corresponding to inter-capillary distances), $\textrm{Pe}$ remains below unity for small molecules.

We further examine the validity of this conclusion for high-MW therapeutics, such as monoclonal antibodies (e.g. Aducanumab, ∼150 kDa), whose diffusivity is substantially reduced. According to the Stokes–Einstein relation, the diffusivity of a solute scales inversely with its hydrodynamic radius $R_h$,

7 $$\begin{aligned} \tilde{D} \propto \frac{1}{R_h}. \end{aligned}$$

For globular proteins, the hydrodynamic radius is commonly approximated to scale with the cube root of molecular weight, $R_h \propto \textrm{MW}^{1/3}$. Combined with the Stokes–Einstein relation, $\tilde{D} \propto 1/R_h$, this yields the commonly used diffusivity scaling [38]:

8 $$\begin{aligned} \tilde{D} \propto \textrm{MW}^{-1/3}. \end{aligned}$$

which we adopt here as an approximate molecular-size scaling for interstitial transport [39, 40]. While alternative empirical correlations exist, this form provides a physically grounded estimate suitable for the comparative analysis performed. Comparing a small-molecule therapeutic (Donepezil, ∼379 Da) with an antibody (Aducanumab, ∼145 kDa) gives a molecular-weight ratio of approximately $3.8\times 10^2$, corresponding to a diffusivity reduction by a factor of ∼7 under this scaling.

Consistent with the experimentally derived values used in this study, the effective diffusivity of Aducanumab is approximately $\tilde{D}^{\mathrm {*}}\approx 30~\mu \textrm{m}^2/\textrm{s}$. Re-evaluating the Péclet number for the antibody case using the same conservative velocity and length scales yields

9 $$\begin{aligned} \textrm{Pe}_{\textrm{Adu}} \approx \frac{(0.1~\mu \mathrm {m/s})(10~\mu \textrm{m})}{30~\mu \textrm{m}^2/\textrm{s}} \approx 3.3\times 10^{-2}\ll 1. \end{aligned}$$

Even for the largest therapeutic considered in this study, transport in the neuropil remains diffusion-dominated. This simplification allows geometric hindrance induced by DS microdomains to be isolated without the confounding influence of pressure-driven flow – providing a clear baseline for assessing limits to therapeutic penetration in neurodegenerative tissue.

**Table 2:** Model input parameters and free diffusivities for selected Alzheimer’s disease therapeutics

Symbol	Description	Value (SI)
*Boundary conditions*		
$c_{0,i}$	Imposed source concentration of species *i*	$1.0\times 10^{-2}$ mol/m$^{3}$
$D_{s,i}$	Astrocyte diffusion coefficient for species *i*	$4.0\times 10^{-10}$ m$^{2}/$s
$D_{\textrm{bl},Adu}$	Blood diffusion coefficient: Aducanumab	$5.5\times 10^{-11}$ m$^{2}/$s
*Free diffusivities of Alzheimer’s disease therapeutics*		
$D_{\textrm{Mem}}^{\textrm{free}}$	Free diffusivity of Memantine	$8.19\times 10^{-10}$ m$^{2}/$s
$D_{\textrm{Don}}^{\textrm{free}}$	Free diffusivity of Donepezil	$6.38\times 10^{-10}$ m$^{2}/$s
$D_{\textrm{Adu}}^{\textrm{free}}$	Free diffusivity of Aducanumab$^\text {a}$	$6.34\times 10^{-11}$ m$^{2}/$s

$$^\text {a}$$ From [40]

### Finite Element Model

All variables in this section are dimensional and correspond directly to the quantities solved numerically; tildes are omitted for clarity. Directly from Eq. 4, diffusive transport with mass conservation in the ECS yields:

10 $$\begin{aligned} \boxed {\varepsilon _{\textrm{p}}\,\frac{\partial c_i}{\partial t} + \nabla \cdot \textbf{J}_i = 0} \end{aligned}$$

with $\varepsilon _{\textrm{p}}$ as the ECS porosity (Table 1), which is spatially uniform. Binding, intrinsic reactions, and volumetric sources are neglected. We model each transported species *i* in the extracellular space (ECS) with a flux:

11 $$\begin{aligned} \textbf{J}_i = -\,{D}_i^{\mathrm {*}} \nabla c_i, \end{aligned}$$

where $c_i$ is the solute concentration and ${D}_i^{\mathrm {*}}$ is the effective diffusivity in the porous ECS for each species.

#### Transport in perivascular Fluid

For luminal or perivascular subdomains:

12a $$\begin{aligned} \frac{\partial c_i}{\partial t} + \nabla \cdot \textbf{J}_i&= 0, \end{aligned}$$

12b $$\begin{aligned} \textbf{J}_i&= -D_{\textrm{bl},i}\,\nabla c_i, \end{aligned}$$

where $D_{\textrm{bl},i}$ is the blood diffusion coefficient from Table 2. Namely, Eqs. 12a and 12b capture the general continuity equation for mass conservation and Fick’s First Law.

##### Thin Astrocytic Diffusion Barrier

Transport across the astrocytic endfoot layer is represented by a thin-layer interface condition:

13 $$\begin{aligned} -\textbf{n}\cdot \textbf{J}_{i,u}= &   \frac{D_{s,i}}{d_s}\big (c_{i,d}-c_{i,u}\big ),\nonumber \\ -\textbf{n}\cdot \textbf{J}_{i,d}= &   \frac{D_{s,i}}{d_s}\big (c_{i,u}-c_{i,d}\big ) \end{aligned}$$

Here $D_{s,i}$ is the astrocytic barrier diffusivity within the thin barrier of thickness $(d_s = th_{as}$). Flux continuity across the interface both in the upper (subscript u) and lower (subscript d) boundaries is enforced.

##### Concentration and Side Boundary Conditions

$$ c_i=c_{0,i} \quad \text {on the para-arterial interface.} $$

Symmetry is imposed on all outer triangular boundaries:

14 $$\begin{aligned} -\textbf{n}\cdot \textbf{J}_i = 0 \end{aligned}$$

which corresponds to domain tessellation of the hexagonal scaffold.

##### Dead Space (DS) Boundaries

15 $$\begin{aligned} -\textbf{n}\cdot \textbf{J}_i = 0 \quad \text {on DS walls}, \quad -\textbf{n}\cdot \textbf{J}_{i,u} = -\textbf{n}\cdot \textbf{J}_{i,d} = 0 \end{aligned}$$

More clearly, the Neumann condition in Eq. 15 indicates that no solute crosses the boundary of the dead space and that both the upper and lower boundaries of each dead space have no out-of-plane (*z*-direction) leakage under the quasi-2D domain assumption. Consistent with experimental observations of hindered molecular transport, DS surfaces are modeled as rigid, non-binding, impermeable structures [12, 13]. This assumption enables isolation of purely geometric hindrance effects without introducing poorly constrained permeability or binding parameters. Impermeability isolates the maximum geometric hindrance attributable to cul-de-sac obstructions; partial permeability would reduce the delays reported here.

### Pharmaceutical Analysis

For the pharmaceutical analysis, the diffusion parameters for three AD therapeutic molecules have been sourced from the literature and are shown in Table 2: A therapeutic from each drug class – NMDA antagonist (Memantine), AChEI (Donepezil) and monoclonal antibody (Aducanumab) – was chosen to inform our analysis. The free diffusivities for Memantine and Donepezil were derived from the Stokes-Einstein equation [39] and compared to effective diffusivity measurements of Donepezil to ensure pharmaceutical validity. $D_{\textrm{bl},B} = D_{\textrm{bl},Don} = D_{\textrm{bl}, Mem}$ as a realistic approximation for small molecules. $D_{\textrm{bl},\text {Adu}}$ was obtained via a viscosity correction of $D_{\textrm{Adu}}^{\textrm{free}}$ with $D \propto \frac{T}{\eta }$, and $\eta _{\textrm{plasma},310K} = 1.1{-}1.2\ \mathrm {mPa\cdot s}$, where T is the temperature and η is the dynamic viscosity used for Stokes-Einstein scaling.

**Fig. 3:**
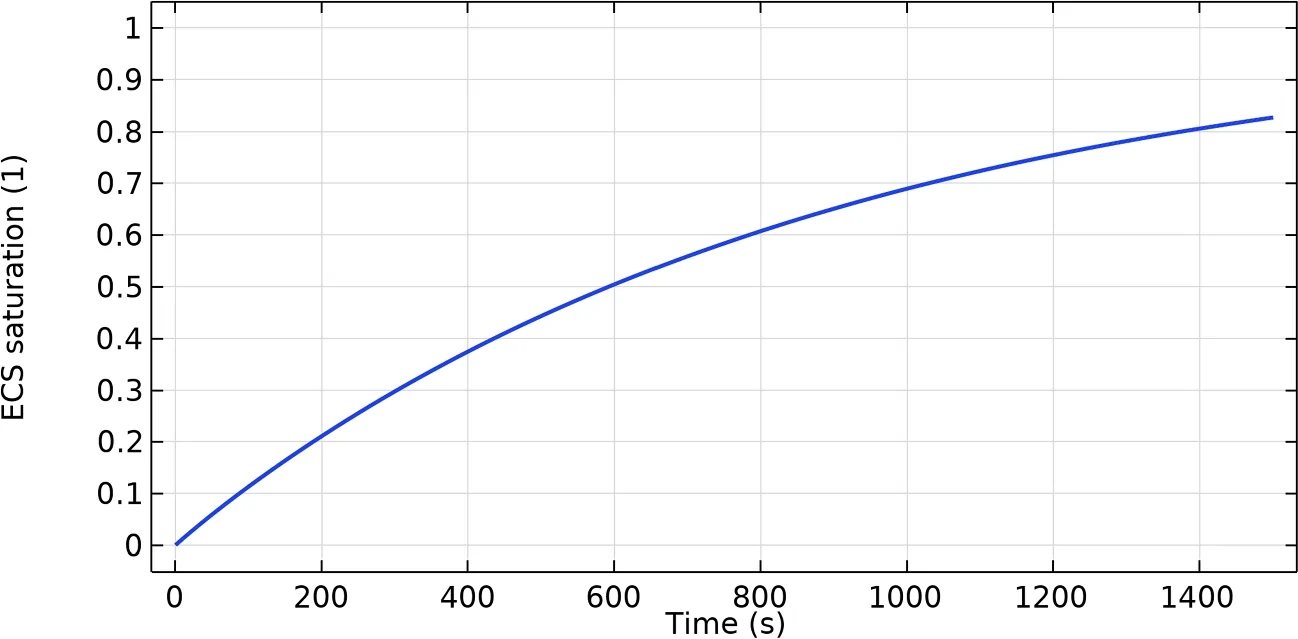
Tracer B domain-integrated normalized uptake $S_B(t)$ under diffusion-only transport in the baseline (no DS) geometry. The half-filling time, which matches experimental observations, is shown

### Temporal Metrics

In Section 3 (Results), temporal transport metrics quantify the timescales over which solutes enter, penetrate and spread through the ECS. We employ two complementary temporal measures that distinguish between global uptake and local penetration.

#### (i) Domain-integrated Uptake (global saturation)

Global ECS uptake is quantified using a normalized saturation metric,

12f $$\begin{aligned} S_i(t) = \frac{1}{c_{0,i}\,A_{\textrm{ECS}}} \int _{\Omega _{\textrm{ECS}}} c_i(x,y,t)\,\textrm{d}\Omega , \end{aligned}$$

which represents the fraction of solute accumulated in the ECS normalized by $c_0$ and the ECS domain area. The corresponding half-saturation time, $t_{1/2}$, characterizes the overall filling kinetics of the ECS and is defined as $S_i(t_{1/2})= 0.5$: the time point at which the mean concentration over the entire ECS reaches $0.5c_0$. While informative of total uptake, this metric does not resolve spatial heterogeneity and may obscure delayed exposure in distal or obstructed regions.

#### (ii) Venule Isoconcentration Arrival Time (penetration delay)

To address the limitations of global saturation and quantify propagation of therapeutically relevant concentrations, we define the venule arrival time $t_{0.5c_0}$ as the first instance in which the isoconcentration at the venular boundary reaches $=0.5c_0$, providing a pathway-limited measure of downstream exposure that is distinct from domain-averaged uptake. As such, $t_{0.5c_0}$ serves as an indicator for the onset of substantial tissue exposure, as a prerequisite for therapeutic effect.

## Results

Where appropriate, results are expressed in dimensionless form to isolate governing transport mechanisms and enable comparison across different extracellular environments. This approach allows transport behavior to be assessed independently of absolute parameter values, highlighting differences driven solely by alterations in the ECS microstructure.

### Validation Results for Tracer B

Experimental measurements and prior modeling studies indicate that diffusion-dominated ECS transport of small (∼3 kDa, $D^\textrm{free}= 1 \times 10^{-10}m^{2}s^{-1}$) tracers occurs on timescales of several minutes in the absence of pathology. Reported and inferred half-filling times $t_{1/2}$ span a range of approximately 500–600 s depending on geometry and measurement conditions [22, 41]. To ensure that our analysis aligns with this experimental benchmark, we compute the domain-integrated uptake metric using Eq. 12f. From Fig. 3, we obtain a half-filling time $t_{1/2} = 593$ s such that $S_B(t_{1/2}) = 0.5$. Our results demonstrate that a baseline ECS geometry without dead spaces, using a global porosity-tortuosity approximation, reproduces experimentally reported half-filling timescales and provides a consistent reference state for subsequent DS perturbation. This allows us to isolate transport hindrance introduced by DS directly; and to achieve this with significant reductions in computational times.

### Introducing DS Microdomains to Tracer B transport

**Fig. 4:**
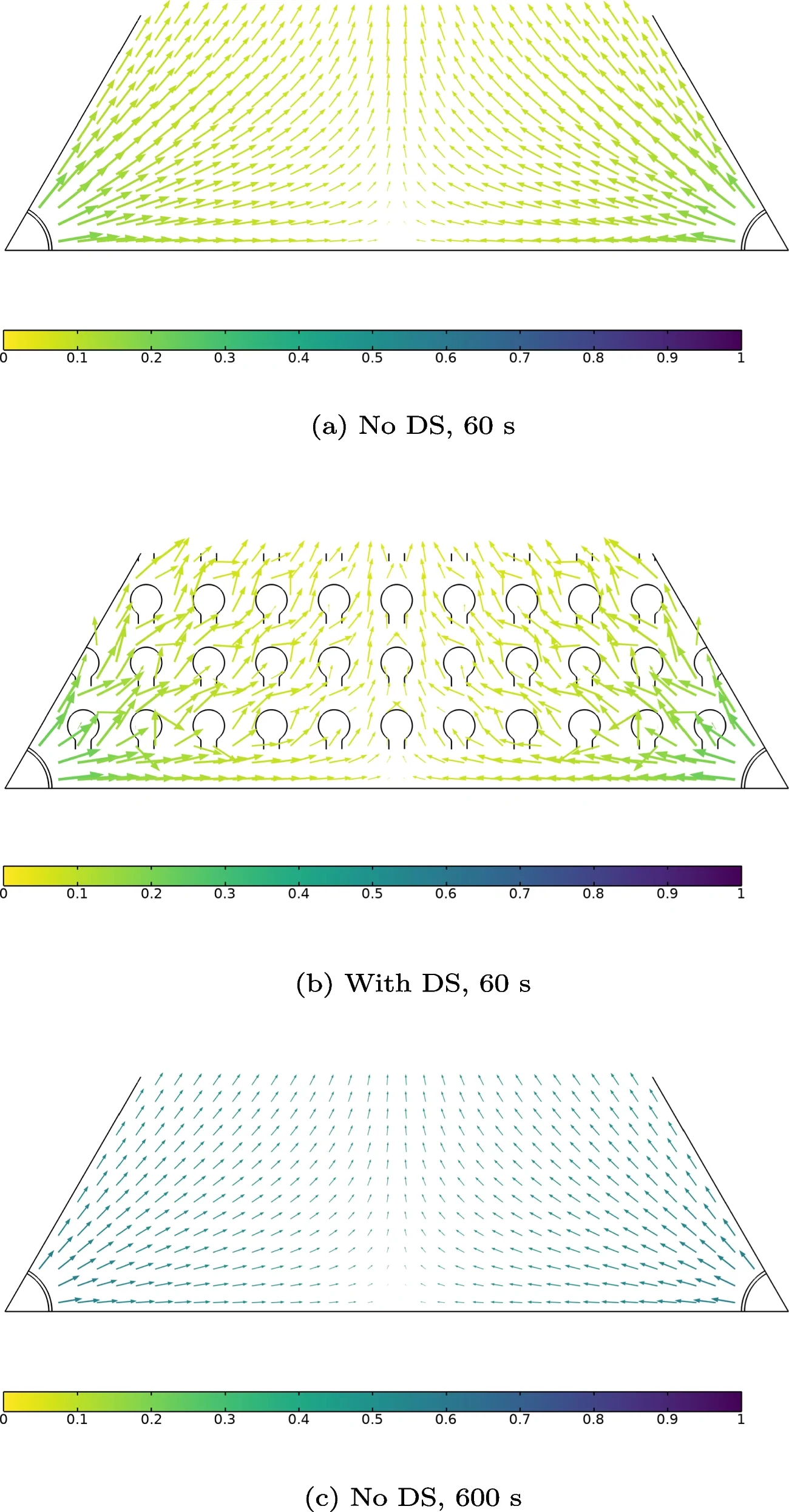

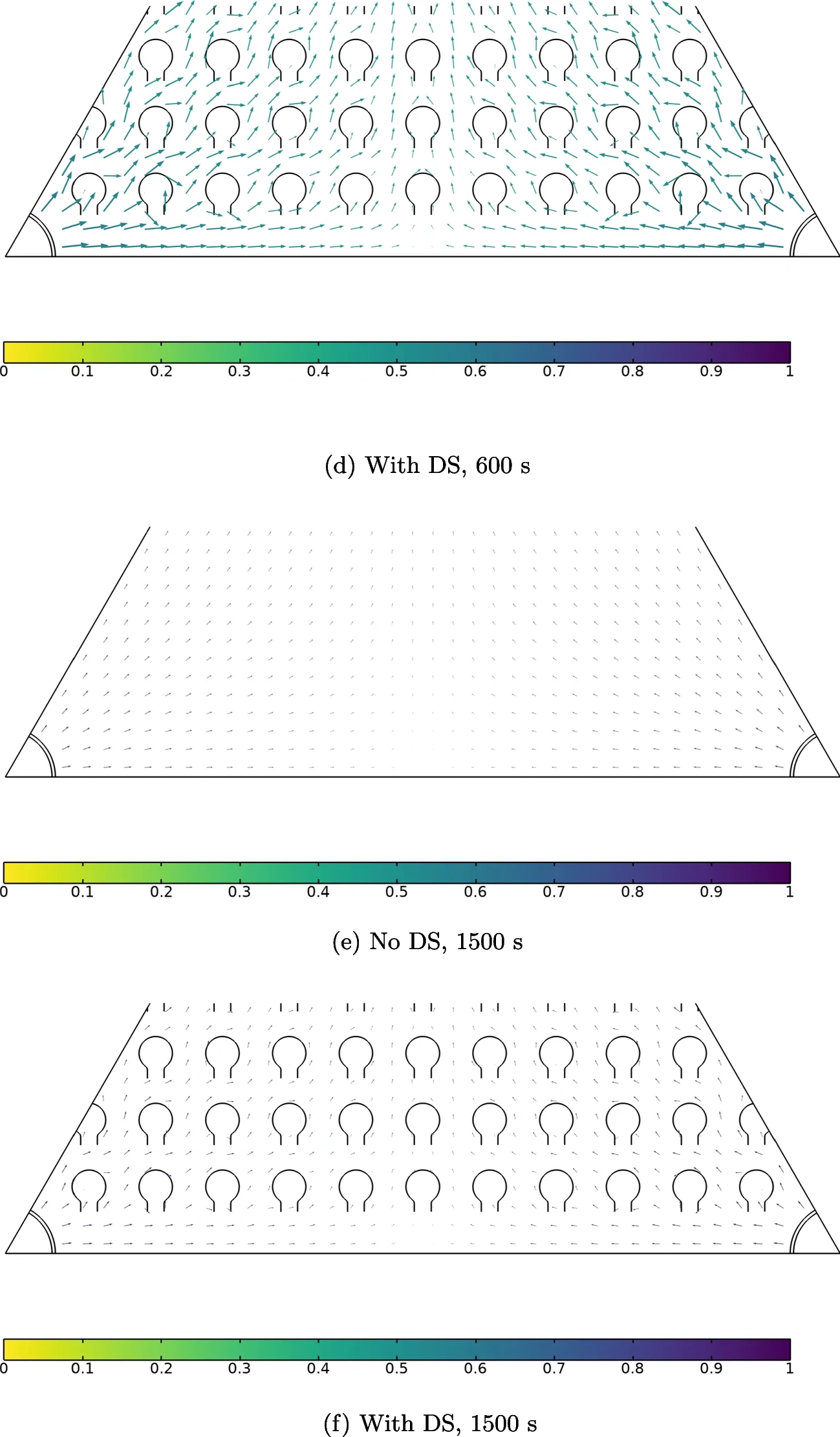
Zoomed-in dimensionless flux vector field visualisations of solute transport at three time points (60 s, 600 s, and 1500 s) across different extracellular space (ECS) configurations for Tracer B. Vector alterations are shown. Concentration gradients decrease rapidly as time progresses

To directly quantify the impact of DS microdomains on diffusion, Fig. 4 presents nondimensional flux vector field visualisations for Tracer B at t=60, 600, and 1500 s. The unobstructed ECS (Fig. 4a ,c , e) displayed radially symmetric dispersion, with flux magnitude decaying away from the arteriole wall. In the DS geometry (Fig. 4b, d, f), flux vectors were locally deflected around impermeable boundaries, creating stagnation zones and reduced gradients that intensified over time.

#### Temporal Results for Tracer B

Temporal metrics capture delays in global uptake, local downstream penetration, and clearance-related persistence. Table 3 summarizes DS-induced delays in bulk filling and downstream exposure.

The introduction of DS microdomains to the Tracer B analysis shifted transport kinetics in a manner that is not fully captured by global uptake. The venule arrival time of the $0.5c_0$ isoconcentration threshold increased from 675 s to 724 s (a 49 s delay), while the domain-integrated half-saturation time increased more modestly (593 to 621 s, a 28 s delay). The larger downstream delay indicates that DS microdomains disproportionately hinder pathway-limited penetration. Given that these delays are incurred within a single representative unit of the cortical scaffold, repeated encounters with DS in a tessellated network are expected to accumulate and compress the effective therapeutic window.

**Table 3:** Effect of dead-space (DS) microdomains on Tracer B transport. Comparison of domain-integrated uptake and pathway-limited venular exposure

Metric	No DS	With DS	Delay
$t_{1/2}$ (Domain half-saturation) [s]	593	621	28
$t_{0.5c_0}$ (Venule arrival) [s]	675	724	49

**Fig. 5:**
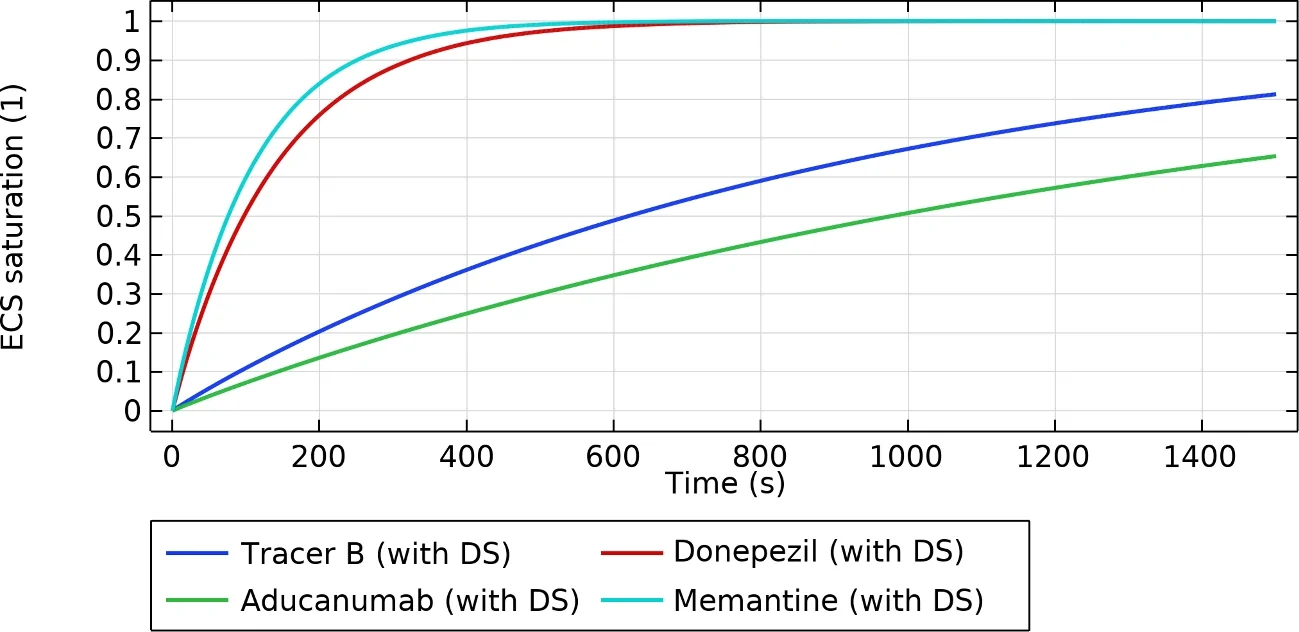
ECS saturation in the presence of DS for all molecules considered

### Pharmaceutical Modeling

Figure 5 shows the saturation curves for each pharmaceutical in the presence of DS. Cumulative ECS uptake curves demonstrated clear reductions in the rate of ECS accumulation for species diffusing within the DS geometry. The temporal metrics calculated from the analysis are shown in Tables 4 and 5.

For all three therapeutics, the DS penalty is larger for the pathway-limited metric $t_{0.5c_0}$ than for the domain-integrated half-saturation time $t_{1/2}$ demonstrating that DS preferentially impede downstream penetration rather than bulk filling. This reflects the inverse scaling of diffusive timescales with $D_i^{\textrm{free}}$, whereby a fixed geometric obstruction imposed by DS microdomains produces progressively larger absolute delays for slower-diffusing solutes, even as the relative perturbation to domain-integrated uptake remains small. The pathway-limited metric $t_{0.5c_0}$ exhibits larger increased absolute delays with decreasing $D_i^{\textrm{free}}$ (Table 5), highlighting the heightened sensitivity of distal exposure at the venule to molecular diffusivity.

**Table 4:** Global ECS half-saturation times with and without dead-space (DS) microdomains

Solute	$\boldsymbol{D_i^{\textrm{free}}}$ [m$^2$/s]	$\boldsymbol{t_{1/2}}$ **[s]**	$\boldsymbol{t_{1/2}}$ **(DS) [s]**	Delay [s]
Memantine	$8.19\times 10^{-10}$	73	76	3
Donepezil	$6.38\times 10^{-10}$	93	98	5
Aducanumab	$6.34\times 10^{-11}$	935	979	44

## Discussion

Our computational framework captures how dead-space (DS) microdomains hinder solute transport in the cerebral extracellular space (ECS), allowing local entrapment and stagnation to emerge directly from the governing equations. Relative to prior finite element studies of ECS transport, which often required $4.5\times 10^5$ to $1\times 10^6$ elements [22] and multi-hour runtimes, our simulations exploit geometric symmetry to obtain grid-independent solutions using $\sim 3.2\times 10^4$ elements with typical runtimes of 5–7 minutes. This represents an $\mathcal {O}(10$–30)× reduction in degrees of freedom and a practical reduction from hours to minutes per parameter set, enabling systematic screening across multiple solutes and microstructural conditions during pharmaceutical development or deployment. Given that baseline porous-medium parameters ($\varepsilon _p=0.2$, λ=1.6) are held fixed across all cases, the observed deviations can be attributed directly to the explicit introduction of impermeable DS boundaries rather than effective transport coefficients [24, 42].

A key outcome of the study is that DS induced hindrance manifests differently in domain-averaged versus pathway limited metrics. For Tracer B, DS increased the domain-integrated half-saturation time from $t_{1/2}=593$ s to 621 s (+28 s), while the downstream venule arrival time of the therapeutically relevant threshold increased from $t_{0.5c_0}=675$ s to 724 s (+49 s). The larger relative penalty in $t_{0.5c_0}$ indicates that DS microdomains disproportionately hinder penetration along constrained routes, even when global uptake remains comparatively stable. This divergence demonstrates how bulk filling timescales can mask distal underexposure: two tissues can exhibit similar domain-averaged uptake while differing materially in downstream availability.

**Table 5:** Arrival time of the $0.5c_0$ concentration contour at the venous outlet, quantifying pathway-limited penetration with and without dead-space (DS) microdomains

Solute	$\boldsymbol{D_i^{\textrm{free}}}$ [m$^2$/s]	$\boldsymbol{t_{0.5c_0}}$ [s]	$\boldsymbol{t_{0.5c_0}}$ (DS) [s]	Delay [s]
Memantine	$8.19\times 10^{-10}$	78	89	11
Donepezil	$6.38\times 10^{-10}$	100	114	14
Aducanumab	$6.34\times 10^{-11}$	1064	1144	80

The pharmaceutical results reinforce this decoupling and demonstrate a consistent size-dependent amplification of DS effects. Across the three therapeutics, DS increased the global half-saturation time by +3 s for Memantine (73→76 s), +5 s for Donepezil (93→98 s), and +44 s for Aducanumab (935→979 s). The pathway-limited venule arrival penalties showed a clearer escalation with decreasing diffusivity: $t_{0.5c_0}$ increased by +11 s for Memantine (78→89 s), +14 s for Donepezil (100→114 s), and +80 s for Aducanumab (1064→1144 s). This provides a concrete mechanistic basis for therapeutic failure modes in neurodegenerative tissue: successful BBB traversal and measurable bulk exposure do not guarantee timely delivery to distal parenchymal targets when cul-de-sac regions are present.

It is important to recognize that drug transport in the brain does not occur within a single isolated microdomain, but rather along extended extracellular pathways that span hundreds of micrometers to millimeters, connecting vascular entry points to cellular targets. Along these trajectories, solutes encounter multiple structural heterogeneities, and in the diffusion-dominated regime, transport times scale with the square of the macroscopic distance ($t \propto {L^2}$). As a result, a local pathway-limited delay of $80~\textrm{s}$ measured over a $250~\mu \textrm{m}$ unit propagates nonlinearly across larger tissue depths. When extrapolated to a physiologically relevant distance of $2.5~\textrm{mm}$ (a 10-fold increase), the delay scales by a factor of 100, to 8000 s (2.2 hours). Consequently, the per-unit delays quantified here correspond to downstream arrival times on the order of tens of minutes for small molecules, and potentially several hours for larger therapeutics such as monoclonal antibodies. These timescales compete with known CNS clearance and PK dynamics [43] and are likely compounded as pharmaceutical diffusion progresses through multiple pathways in 3D under stochastic DS clustering.

Memantine and Donepezil are small-molecule agents with elimination half-lives on the order of 60–100 h, supporting sustained systemic and CNS exposure under repeated dosing [44, 45]. Aducanumab exhibits a markedly longer systemic half-life of approximately 25 days and is administered at monthly intervals [46]. While prolonged PK persistence enables sustained systemic availability, it does not guarantee uniform distribution within the brain parenchyma in the presence of DS microdomains – as spatial concentration gradients may persist even under steady-state conditions. Pathway-limited delays reflect underlying structural barriers that can lead to chronically underexposed regions despite prolonged systemic exposure. The reported delays determine the timing and spatial extent of local target engagement rather than the total duration of systemic drug availability.

Beyond absolute delay, the results also have implications for threshold-dependent drug action. The spatial heterogeneity introduced may result in inconsistent target engagement by shaping spatial concentration profiles that govern target occupancy. Many CNS therapeutics require concentrations to exceed a minimum effective threshold (receptor occupancy or plaque-binding levels) to achieve pharmacological effect. The pathway-limited metric $t_{0.5c_0}$ can be interpreted as a proxy for the time required to reach such thresholds in distal regions. DS-induced delays therefore do not simply shift concentration profiles in time, but may determine whether specific regions ever reach therapeutically relevant levels within the available exposure window. This effect is particularly pronounced for diffusion-limited compounds, where delayed arrival combined with systemic clearance may result in persistent sub-threshold exposure in obstructed regions.

The role of advection [27, 47] was assessed by a nondimensional analysis, which indicated that transport remains diffusion-dominated under plausible parenchymal conditions. The corresponding Péclet estimates support this conclusion: under neurodegenerative conditions away from high-velocity perivascular regions, adding advection does not materially alter the penetration timescales relative to the geometric obstruction effects induced by DS. The results indicate that explicit geometric obstruction is a dominant driver of microscale delay under DS remodeling. This distinction is clinically relevant as it narrows the set of pathophysiological parameters that must be constrained to generate actionable predictions. More broadly, the work supports the interpretation that models calibrated only to global filling timescales (e.g., $t_{1/2}$) may systematically overestimate target engagement in plaque-rich or DS-dense tissue.

Beyond the present AD application, the computational efficiency of the framework makes it well suited for several CNS drug-delivery problems. In particular, it can be used for rapid screening of candidate therapeutics with different diffusivities; for comparing the penetration of small molecules, antibodies, and nanoparticle-assisted formulations in remodeled ECS environments; and for evaluating how local microstructural obstructions alter downstream target exposure. Future extensions will incorporate stochastic DS distributions and clustering to assess whether obstruction effects become nonlinear in plaque-dense regions. Such clustering is clinically plausible and could produce contiguous transport barriers whose impact exceeds the sum of isolated obstructions. Including these features would support patient-specific stratification strategies [48], where imaging of ECS integrity or DS burden could inform dosing strategies or delivery selection.

## Conclusion

This study introduces a validated spatiotemporal framework that explicitly incorporates DS microdomains into an anatomically grounded scaffold of the ECS. By embedding DS as impermeable obstructions, we demonstrate how microstructural anomalies generate diffusion hindrance, saturation delays, and solute entrapment – effects that scale nonlinearly with molecular weight and diffusivity. Importantly, by introducing two complementary metrics, we show that DS microdomains can preserve agreement with experimental bulk diffusion timescales while simultaneously masking a hidden layer of spatially heterogeneous underexposure. Our approach integrates: (i) anatomically grounded representations under neurodegenerative remodeling, with (ii) pharmaceutical considerations, iii) multiphysics coupling with an experimental validation, and (iv) targeted nondimensional analyses that reveal underlying transport mechanisms. A nondimensional analysis is also employed in the interpretation of results, enhancing the generalizability of the framework across multiple pharmaceuticals and dosages. We achieved high numerical fidelity while operating with low runtimes, enabling systematic exploration of diffusion hindrance by pathological microstructures during pharmaceutical development. Future extensions will incorporate stochastic DS arrangements and dynamic physiological conditions. Clinical trial stratifications may benefit from considering ECS integrity and DS density as biomarkers for drug penetrability, thus moving toward more personalized dosing strategies in Alzheimer’s disease.

## Figures and Tables

**Fig. 6:**
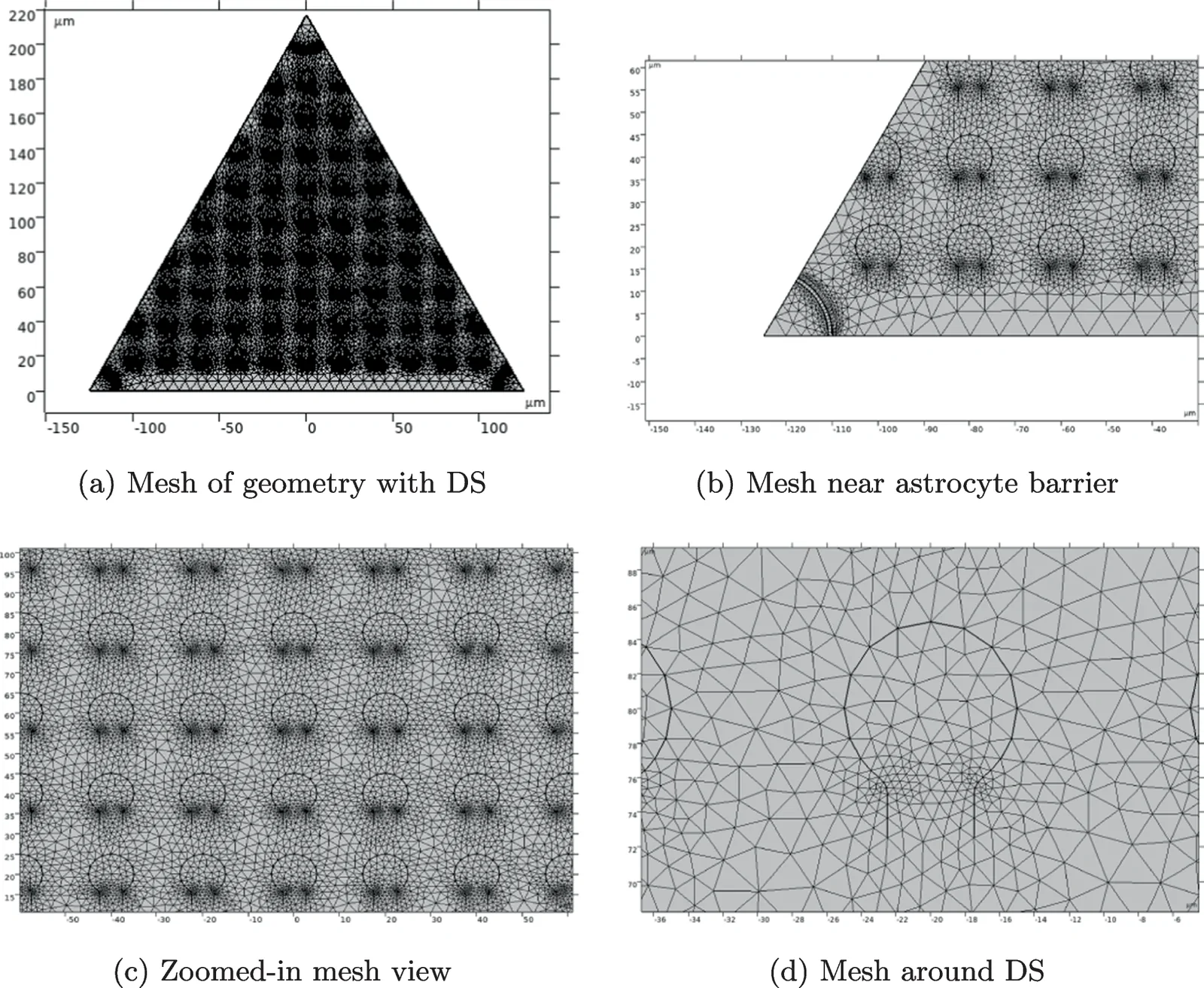
Mesh configuration used in the simulations. Panels shown top to bottom: (**a**) overall mesh structure; (**b**) near astrocyte barrier; (**c**) zoomed-in view; (**d**) mesh around DS

**Fig. 7:**
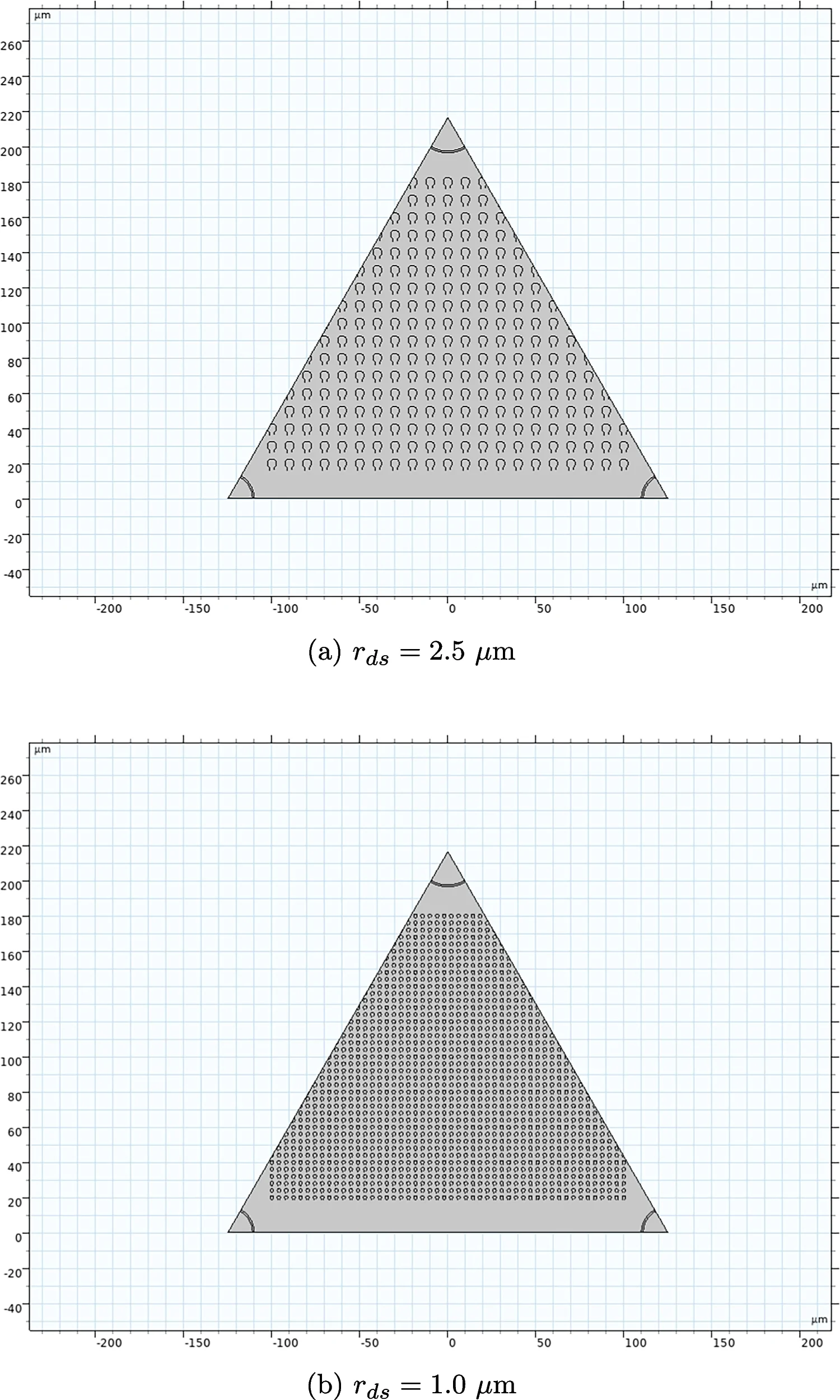
Dead-space (DS) geometries for decreasing characteristic radii, showing the transition from mesoscale obstructions to sub-micron features

**Fig. 8:**
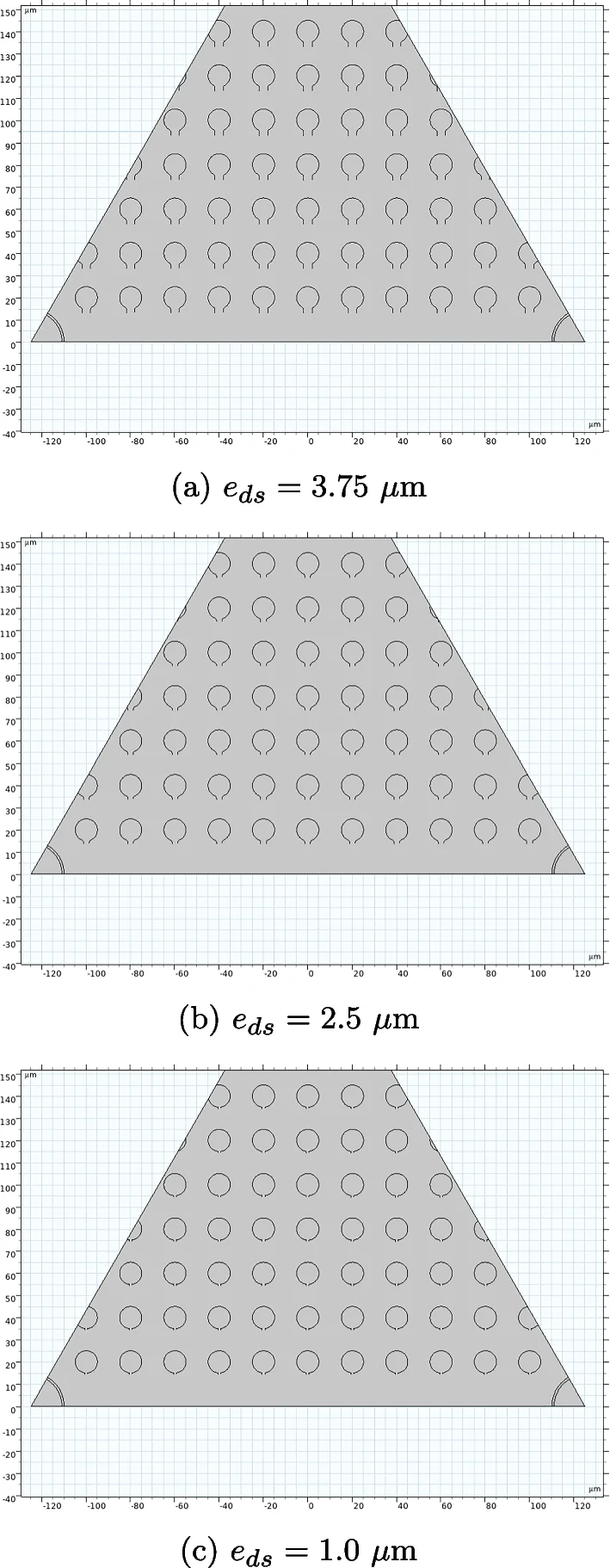
Dead-space (DS) geometries for decreasing entrance sizes

## Appendix A: Meshing and Convergence

### A.1: Numerical Method and Mesh Setup

The governing transport equations were solved numerically using the finite element method in COMSOL Multiphysics$^{\circledR}$. The computational domain was discretized using an unstructured triangular mesh generated by COMSOL’s built-in meshing algorithms (see Fig. 6). To resolve steep gradients whilst maintaining computational efficiency, the final mesh consisted of approximately $3.2 \times 10^4$ elements, with a minimum element size of 0.5 µm and a maximum of 25 µm. The simulations were executed on a workstation equipped with a 13$^{\text {th}}$ Gen Intel$^{\circledR }$ Core i9–13950HX processor and 32 GB of RAM; with typical run times of 5-7 minutes. For reference, the simulations by Jin et al [22] contained $4.5 \times 10^5$ to $1 \times 10^6$ elements and ran for 1-12 hours.

### A.2: Grid Convergence Verification

To quantify the discretisation error of the numerical solution, a grid convergence study was performed. The Grid Convergence Index (GCI) – a standardised method based on Richardson Extrapolation – was used to estimate the error margin. This procedure requires simulations on a minimum of three successively refined meshes (coarse, medium, and fine) to reliably estimate the asymptotic convergence range. The first step in the Richardson Extrapolation procedure is to calculate the representative cell length, *h*, for each of the three mesh levels. Given that the elements are non-uniform in 2D, the representative element’s edge length *h* is calculated using the equation:

$$ h=\left( \frac{1}{N} \sum _{i=1}^{N} \Delta S_i \right) ^{1/2} $$

where $\Delta S_i$ represents the surface of the element. The representative element length for each element is therefore given by: $h_{\text {coarse}}$, $h_{\text {medium}}$, and $h_{\text {fine}}$. To verify that the refinement levels are adequate, the refinement ratios between adjacent mesh levels is calculated as follows:

$$ r_{32}=\frac{h_{\text {coarse}}}{h_{\text {medium}}}, \quad r_{21}=\frac{h_{\text {medium}}}{h_{\text {fine}}} $$

Having generated three different meshes with suitable refinement ratios, a simulation was performed with each mesh, and the results were recorded for analysis. This approach ensures that the extrapolation and refinement study is both accurate and reliable. To quantify the solution differences between the fine and medium meshes ($\epsilon _{21}$), as well as between the medium and coarse meshes ($\epsilon _{32}$), these differences were calculated:

$$\begin{aligned} \epsilon _{21}= &   \phi _2 - \phi _1\\ \epsilon _{32}= &   \phi _3 - \phi _2 \end{aligned}$$

With the parameter *s* describing whether the convergence is monotonic (s=1) or oscillatory (s=-1):

$$ s=\text {sign}\left( \frac{\epsilon _{32}}{\epsilon _{21}}\right) = 1 $$

The non-linear equation is solved by applying a root-finding algorithm, thereby enabling the determination of the order of convergence.

$$ \frac{1}{\ln (r_{21})}\left| \ln \left| \frac{\epsilon _{32}}{\epsilon _{21}}\right| + \ln \left| \frac{r_{21}^p - s}{r_{32}^p - s}\right| \right| - p = 0 $$

where $$q = \ln \left( \left| \frac{\epsilon _{21}}{\epsilon _{32}}\right| \right) $$ and $$s = \text {sign}\left( \frac{\epsilon _{32}}{\epsilon _{21}}\right) $$.

With the order of convergence calculated, the solution on an infinitely fine mesh, $\phi _0$, can be determined by:

$$ \phi _0 = \phi _1 + \frac{\phi _1 - \phi _2}{r_{21}^p - 1} = 82.7487 $$

where $\phi _1$, $\phi _2$, and $\phi _3$ correspond to the fine mesh, medium mesh, and coarse mesh, respectively, with values of $\phi _1 = 82.7290$, $\phi _2 = 82.7100$, and $\phi _3 = 82.7230$, representing the saturation of Tracer B (%) at t=1500 s. The relative error and relative Grid Convergence Index (GCI) are subsequently computed as follows:

$$\begin{aligned}&e_{21} = \left| \frac{\phi _2 - \phi _1}{\phi _1} \right| = 0.02\%\\&GCI_{21} = \left| \frac{e_{21}}{(r_{21}^p - 1)} \right| = 0.03\% \end{aligned}$$

Based on these results, the finest mesh generates grid-independent results which fall within acceptable limits.

### Analysis of the Zero-Flux Condition

The zero-flux condition, expressed as the homogeneous Neumann boundary condition, is fundamental for modeling impermeable surfaces. It is mathematically described as:

$$ - \textbf{n} \cdot \textbf{J}_i = 0 $$

where $\textbf{n}$ is the unit vector normal to the surface and $\textbf{J}_i$ is the flux density of species *i*. This condition indicates that there is no mass transfer across the boundary surface, as the volume of the dead space is impermeable.

The enforcement of the zero-flux condition on the walls of the dead spaces was implemented using a strong, direct approach. Specifically, the walls of the dead spaces were defined as distinct boundary entities with their own outward normals. This methodology ensures that the condition is applied directly to the boundary surfaces, without requiring additional techniques to manage flow continuity at nodes shared between different elements. Flow continuity at non-internal interfaces was ensured through the standard properties of the finite element solver. This direct method is methodologically superior to the penalty method, which is a weak enforcement that may introduce errors or require fine-tuning of a penalty coefficient (α) to achieve the desired accuracy.

## Appendix B: Sensitivity Analysis of Dead-Space Geometry

### B.1: Motivation

To quantify the impact of geometric assumptions on transport hindrance, we performed a sensitivity analysis on the characteristic size of dead-space (DS) microdomains. Specifically, we assessed whether the transport delays observed in the baseline configuration persist as the obstruction length scale is reduced, whether by reducing the DS radius or DS entrance size.

### B.2: Methodology: DS Radius

The analysis was conducted for Aducanumab, the largest and most diffusion-limited therapeutic considered in this study. The DS radius was varied (Fig. 7) from the baseline value $r_{ds} = 5.0~\mu \textrm{m}$ to smaller scales of $2.5~\mu \textrm{m}$ and $1.0~\mu \textrm{m}$ while all other geometric and transport parameters, including DS volume fraction, were held constant.

### B.3: Methodology: DS Entrance Size

In a complementary analysis, the characteristic size of the DS entrance, ${e_{ds}}$, was varied (Fig. 8) independently of the pocket radius to isolate the effect of access resistance on transport.

### B.4: Results: DS Radius

Table 6 summarizes the effect of DS radius on both global uptake ($t_{1/2}$) and pathway-limited penetration ($t_{0.5c_0}$).

**Table 6:** Sensitivity of Aducanumab transport metrics to DS radius

Case	$\boldsymbol{r_{ds}}$ [μm]	$\boldsymbol{t_{1/2}}$ (DS) [s]	$\boldsymbol{t_{0.5c_0}}$ (DS) [s]
DS (baseline)	5.0	979	1144
Reduced DS	2.5	966	1122
Micro DS	1.0	959	1114
No DS	–	935	1064

### B.5: Results: DS Entrance Size

Table 7 summarizes the effect of DS entrance size on both global uptake ($t_{1/2}$) and pathway-limited penetration ($t_{0.5c_0}$).

**Table 7:** Sensitivity of Aducanumab transport metrics to DS radius

Case	$\boldsymbol{e_{ds}}$ [μm]	$\boldsymbol{t_{1/2}}$ (DS) [s]	$\boldsymbol{t_{0.5c_0}}$ (DS) [s]
DS (baseline)	5.0	979	1144
Reduced entrance	3.75	973	1131
Reduced entrance 2	2.5	971	1126
Reduced entrance 3	1.25	967	1123
No DS	–	935	1064

### B.6: Discussion

Reducing the DS radius produces a monotonic decrease in transport delay, with both $t_{1/2}$ and $t_{0.5c_0}$ approaching the no-DS baseline as the obstruction scale decreases. Reducing the entrance size produces a comparatively smaller reduction in transport delay than changes to the DS radius, suggesting that cavity geometry contributes more strongly to hindrance than access resistance. Capturing sub-micron features requires finer discretization to resolve steep concentration gradients near boundaries, which limits the practical scalability of high-resolution simulations for large parameter sweeps or pharmaceutical screening applications.

### B.7: Implications

From a modeling standpoint, there is a trade-off between physical fidelity and computational tractability. While resolving smaller DS features increases biological realism, it rapidly leads to computational costs that may require high-performance computing resources. Therefore, mesoscale abstractions such as the baseline $r_{ds}=5~\mu \textrm{m}$ case, provide a practical and computationally efficient upper-bound estimate of obstruction-induced transport delays, while sensitivity analyses such as the present study ensure that conclusions remain robust across physiologically relevant length scales.

## Appendix C: Dimensional Analysis

### C.1: Characteristic Scales and Dimensionless Variables

To normalize Eq. 1, we introduce characteristic scales based on the domain geometry and physicochemical properties of the solute. The relationship between a dimensional variable $\tilde{y}$ and its dimensionless counterpart *y* is

12g $$\begin{aligned} y = \frac{\tilde{y}}{\tilde{Y}_{\textrm{scale}}}. \end{aligned}$$

**Table 8:** Definition of dimensional variables, characteristic scales, and dimensionless variables

Physical quantity	Dimen-sional variable	Charac-teristic scale	Scale definition	Dimen-sionless variable
Concentration	$\tilde{c}$	$\tilde{c}_0$	Source concentration at arteriole	*c*
Length	$\tilde{\textbf{x}}$	$\tilde{L}_c$	Characteristic domain length	$\textbf{x}$
Time	$\tilde{t}$	$\tilde{t}_c$	$\tilde{L}_c^2/\tilde{D}^{\textrm{free}}$	*t*
Velocity	$\tilde{\textbf{u}}$	$\tilde{U}_c$	Characteristic interstitial velocity	$\textbf{u}$
Diffusivity	$\tilde{D}$	$\tilde{D}^{\textrm{free}}$	Free aqueous diffusivity	*D*
Operator	$\tilde{\nabla }$	$1/\tilde{L}_c$	Inverse length	∇

### C.2: Dimensionless groups

Substituting the characteristic scales from Table 8 into Eq. 1 yields dimensionless groups that characterize the relative importance of transport mechanisms.

**Table 9:** Key dimensionless numbers governing solute transport in the ECS

Dimensionless number	Symbol	Definition	Physical interpretation
Péclet number	$\textrm{Pe}$	$\frac{\tilde{U}_c \tilde{L}_c}{\tilde{D}^{\textrm{free}}}$	Advection vs diffusion
Tortuosity	λ	—	Geometric hindrance ($\tilde{D}^{*}=\tilde{D}^{\textrm{free}}/\lambda ^2$)
Porosity	ε	—	Total solute capacity

### C.3: Dimensionless Boundary Conditions

Boundary conditions transform naturally under the same scaling and are summarized in Table 10.

**Table 10:** Transformation of boundary conditions

Boundary location	Physical condition	Dimensional form	Dimensionless form
Arteriole wall	Constant source	$\tilde{c}=\tilde{c}_0$	c=1
Dead-space wall	Impermeable	$\textbf{n}\cdot \tilde{\nabla }\tilde{c}=0$	$\textbf{n}\cdot \nabla c=0$
Symmetry plane	No flux	$\textbf{n}\cdot \tilde{\nabla }\tilde{c}=0$	$\textbf{n}\cdot \nabla c=0$
Astrocytic barrier	Flux continuity	$-\textbf{n}\cdot \tilde{\textbf{J}}=\tilde{P}_{\textrm{barrier}}(\tilde{c}_{\textrm{out}}-\tilde{c}_{\textrm{in}})$	$-\textbf{n}\cdot \textbf{J}=\textrm{Sh}(c_{\textrm{out}}-c_{\textrm{in}})$

Here $\textrm{Sh}$ denotes the Sherwood number, representing the dimensionless mass-transfer coefficient across the astrocytic barrier.
